# Grey and white matter volumes either in treatment-naïve or hormone-treated transgender women: a voxel-based morphometry study

**DOI:** 10.1038/s41598-017-17563-z

**Published:** 2018-01-15

**Authors:** Giancarlo Spizzirri, Fábio Luis Souza Duran, Tiffany Moukbel Chaim-Avancini, Mauricio Henriques Serpa, Mikael Cavallet, Carla Maria Abreu Pereira, Pedro Paim Santos, Paula Squarzoni, Naomi Antunes da Costa, Geraldo F. Busatto, Carmita Helena Najjar Abdo

**Affiliations:** 1 Department of Psychiatry, University of São Paulo Medical School (FMUSP), São Paulo, Brazil; 2 Laboratory of Neuroimage in Psychiatry (LIM 21), Research in Applied Neuroscience, Support Core of the University of São Paulo (NAPNA-USP), São Paulo, Brazil

## Abstract

Many previous magnetic resonance imaging (MRI) studies have documented sex differences in brain morphology, but the patterns of sexual brain differences in transgender women – *male sex assigned at birth* – with a diagnosis of gender dysphoria (TW) have been rarely investigated to date. We acquired T1-weighted MRI data for the following four (n = 80) groups: treatment-naïve TW (TNTW), TW treated with cross-sex hormones for at least one year (TTW), cisgender men, and cisgender women (cisgender individuals as controls). Differences in whole-brain and regional white matter volume and grey matter volume (GMV) were assessed using voxel-based morphometry. We found lower global brain volumes and regional GMVs in a large portion of the posterior-superior frontal cortex in the cisgender women group than in the TTW and cisgender men groups. Additionally, both transgender groups exhibited lower bilateral insular GMVs than the cisgender women group. Our results highlight differences in the insula in both transgender groups; such differences may be characteristic of TW. Furthermore, these alterations in the insula could be related to the neural network of body perception and reflect the distress that accompanies gender dysphoria.

## Introduction

Transgender people (frequently referred to as trans people) experience incongruence between their personal sense of gender identity and their sex assigned at birth^1^. According to the DSM-5 classification, trans people who suffer significant distress associated with their gender perception are diagnosed with gender dysphoria, which is characterized by distress accompanying the incongruence between one’s sex assigned at birth and the gender with which one identifies. Although not all individuals express discomfort as a result of this discordance, many may experience personal anguish if hormone and/or surgical intervention is not available^2^. Considering that the aetiology of gender dysphoria is unknown, it has been postulated that foetal sexual brain differentiation during the second half of pregnancy does not correspond to the development of the rest of the body in transgender people. This assumption implies that neuroanatomical features reflective of the causative processes that determine gender dysphoria may be detectable in the brain^3^.

Many neuroimaging studies to date have investigated sex differences in brain morphology. Initial investigations in this field have demonstrated regional brain volume differences between men and women through magnetic resonance imaging (MRI) based on brain regions of interest (ROIs) defined manually or with semiautomated techniques^4,5^. The introduction of voxel-based morphometry (VBM) has made it feasible to conduct voxel-wise comparisons in an automated fashion across the whole brain. Many subsequent VBM studies have reported findings indicating sexual differences in the human brain regarding the volumes of specific structures^6,7^. In addition, the results of brain volumetric studies have been complemented by MRI studies that have investigated sex differences in regard to measurements of cortical thickness across the whole brain^8,9^.

In a recently published meta-analysis of MRI studies on this subject using VBM or tensor-based morphometry (which also affords measures of regional brain volumes and grey matter density), Ruigrock *et al*. concluded that sexual differences in brain volumes, most notably in the insula (related to human consciousness), limbic structures (emotion, behaviour and motivation, among others), basal ganglia (numerous functions, including control of voluntary motor movements, learning and cognition), thalamus (sensory and motor signals to the cerebral cortex), language-related cortical regions and cerebellum (motor control and cognitive functions), may be detected with MRI^10^. Furthermore, previous VBM studies have also reported findings of white matter volume (WMV) sex differences in the cerebellum, cerebral lobes and corpus callosum^7,11^.

All MRI studies in this field to date have suggested that the sex/gender of the brain does not present such well-defined characteristics presented by the genitalia^12–14^. A recent study showed that this distinction would only be possible if sex/gender differences in brain characteristics were highly dimorphic and if brains were inherently congruent with male or female sex/gender. Although this study noted differences in sex/gender and behaviour, the human brain has a variety of individual characteristics that may each be more masculine or feminine. Consequently, human brains cannot be classified into two distinct categories of “male brain” *versus* “female brain”^15^. Likewise, there is a growing a social trend considering gender as a continuum, rather than distinct male and female genders^16^.

The possible brain anatomical features associated with transgender women − *male sex assigned at birth* − with gender dysphoria diagnosis (TW) have been rarely investigated to date. The first MRI study of TW used a ROI-based method, focusing on the corpus callosum, and did not show differences between TW and cisgender men (CM) or cisgender women (CW) controls^17^. A decade later, one study reported that the corpus callosum pattern in TW was congruent with gender identity^18^. To date, four VBM studies investigating differences in regional grey matter volume (GMV) have compared transgender people with cisgender controls. The first of these studies on adult TW showed that the right putamen had a greater GMV in treatment-naïve TW (TNTW) than in CM^19^. The second study demonstrated greater GMV in the temporo-parietal junction, the inferior frontal cortex and the insular cortex of the right hemisphere in TNTW than in cisgender controls, as well as between-group volumetric differences of the putamen and the thalamus^20^. The third VBM study showed lower regional GMV in the left angular gyrus and the inferior parietal lobule in the transgender group^21^. The fourth study evaluated adolescents and noted lower volume of the cerebellum (bilaterally) and the hypothalamus in transgender girls than in cisgender boys^22^. Two other studies examined cortical thickness measurements in TNTW and demonstrated consistent results, that is, higher cortical thickness in TNTW than in CM controls^23,24^. Although reported findings have been heterogeneous to date, the above VBM studies detected GMV variations in the angular gyrus, the insula and the putamen of TW^19–21^. These brain regions were hypothesized to be relevant in transgender people because they are related to the neural network of body perception^20,21^.

Only one previous VBM study of TNTW has assessed regional WMV and verified that TNTW exhibit lower WMV in the precentral area than CW^20^. On the other hand, the white matter (WM) microstructure of TW has been evaluated using diffusion tensor imaging (DTI) in previous MRI studies. One study showed that the fractional anisotropy of several fasciculi in TNTW was halfway between those of CM and CW controls^25^. Another study did not find differences in fractional anisotropy between TNTW and cisgender controls, although the mean diffusivity in CW and TNTW was higher than that in CM^26^. Overall, these MRI studies have provided some evidence supporting the view that there may be regional GMV and WMV differences between TW and cisgender controls. However, the patterns detected thus far are not clearly associated with female brain characteristics, thus raising the possibility that TW-related brain volume differences are not necessarily related to signs of brain feminization^27^.

All the abovementioned VBM studies of adult TW were performed before cross-sex hormone treatment (CHT). However, it is important to note that most TW have a history of prolonged use of sex steroids. Three VBM studies have investigated the influence of circulating sex steroids on cisgender individuals and have reported the following findings: (i) sex hormones influence the organization of the grey matter (GM) in cortical regions during adolescence and adulthood^6^, (ii) increased levels of circulating testosterone during puberty in boys are associated with sexual differences in the amygdala and the hippocampus^28^, and (iii), increased levels of 17β-oestradiol in girls are related to sexual differences in GM^29^. Another ROI-based MRI study investigated the influence of CHT for four months in TW and demonstrated the presence of brain volume reductions towards female proportions^30^. In another MRI study, cortical thickness in TW was assessed prior to and after six months of CHT, and evidence that sex steroids are associated with reduced cortical and subcortical structures was reported^31^.

To further analyse possible brain anatomical features associated with TW and the influence of CHT, the present cross-sectional MRI study aimed to investigate differences in whole-brain and regional GMV and WMV by VBM in two TW groups: TNTW and TW after at least one year of CHT (TTW). These TW groups were compared with CM and CW as control groups. Moreover, by applying the small-volume correction (SVC) method, we intended to replicate the findings of previous MRI investigations for the following brain regions: the angular gyrus, the insula and the putamen^19–21^. The corpus callosum, which has also been previously reported to present changes in TW^18^, was additionally assessed by SVC in the current study.

## Results

### Sample characteristics

Table 1 shows the sociodemographic and clinical data for all groups. TW and controls did not differ in age, but the schooling level was significantly lower in TNTW than in controls. The total testosterone level was significantly lower in TTW than in TNTW (p < 0.001), although the oestradiol level was not significantly different between the two groups (p = 0.892). No changes in secondary sexual characteristics, which were evaluated by physical examination, were reported for the TNTW group.

**Table 1:** Sample details regarding demographic and clinical data.

Variables	Treatment- naïve TW N = 20	Hormone-treated TW N = 20	Cisgender men N = 20	Cisgender women N = 20	P-value
Age (years)	27 (9), 18–49	31 (7), 18–46	28 (7), 18–42	30 (9), 18–46	0.144^a^
Education (years)	11 (2), 8–16	12 (3), 8–20	14 (3), 11–22	13 (3), 9 −18	0.033^a,b^
CHT (months)	—	36 (61), 19–252	—	—	—
Left-handed^c^	—	—	1 (5%)	1 (5%)	0.999^d^
Hormonal dosages*					
T (ng/dL)	648.3 (267.4)	65.9 (110.6)	—	—	<0.001^e^
Free T (ng/dL)	391.3 (23.8)	—	—	—	
FSH (IU/L)	7.2 (7.1)	5.9 (11.9)	—	—	<0.001^e^
LH (IU/L)	6.5 (2.7)	2.9 (6.4)	—	—	<0.001^e^
Oestradiol (pg/mL)	30.2 (7.8)	55.8 (93.0)	—	—	0.892^e^

Notes: TW = transgender women diagnosed with gender dysphoria; CHT = cross-sex hormone therapy; T = testosterone; FSH = follicle-stimulating hormone; LH = luteinizing hormone. All variables are reported in means (standard deviations). The respective ranges follow age, education, and CHT. ^a^Results from Kruskal-Wallis tests. ^b^Group differences: treatment- naïve TW < cisgender women (p = 0.024), treatment- naïve TW < cisgender men (p = 0.04); Holm-Šidák (*post hoc* test). ^c^Handedness: number of individuals (percentage), ^d^Fisher’s exact test. *Normal ranges: oestradiol, up to 42.6 pg/mL; LH, 1.7 to 8.6 IU/L; FSH, 1.5 to 12.4 IU/L; T, 249 to 836 ng/dL; and Free T, 131 to 640 pmol/L. **Free T measurement for TW receiving CHT in clinical follow-up is not routine at HC-FMUSP. ^e^Results from Mann-Whitney *U* tests comparing groups: T, FSH and LH were significantly higher in treatment-naïve TW than in hormone-treated TW.

### Imaging data

#### Total brain volumetric differences

The total brain volumes of the four groups are shown in Table 2. There were no significant between-group differences in the total brain volumes between the TW groups. All brain compartments showed reduced global volumes in the CW group compared with the three other groups (Table 2).

**Table 2:** Between-group comparisons of total brain volumes.

Total volumes	Treatment-naïve TW N = 20	Hormone-treated TW N = 20	Cisgender Men N = 20	Cisgender Women N = 20	P-value^a^
GM (mL)	726 (59)	715 (46)	755 (62)	667 (46)	<0.001
CSF (mL)	310 (28)	302 (36)	321 (26)	278 (29)	<0.001
WM (mL)	522 (46)	516 (40)	532 (41)	481 (36)	0.001
TV (mL)	1248 (102)	1232 (83)	1286 (99)	1148 (79)	<0.001
TIV (mL)	1558 (126)	1534 (110)	1608 (117)	1425 (99)	<0.001
**Significant total volume differences in** ***post hoc*** **two-group comparisons** ^**b**^					
	**GM**	**CSF**	**WM**	**TV**	**TIV**
**P-value** ^**b**^					
Treatment-naïve TW > cisgender women	0.01	0.04	0.007	0.002	0.001
Treated TW > cisgender women	0.023	0.02	0.042	0.023	0.014
Cisgender men > cisgender women	<0.001	<0.001	0.002	0.001	0.001

Notes: TW = transgender women diagnosed with gender dysphoria; GM = grey matter; CSF = cerebrospinal fluid; WM = white matter; TV = total tissue volume calculated as GM + WM; TIV = total intracranial volume (GM + WM + CSF). Total volumes are given in means (standard deviations); mL = millilitre. ^a^Results from the ANOVA comparing the four groups. ^b^Verified by Tukey HSD tests.

#### Regional GMV differences

In the whole-brain exploratory analysis, statistical parametric mapping (SPM) for ANCOVA comparing regional GMVs between groups showed one large voxel cluster corresponding to a between-group difference with a peak statistical significance in the left superior medial frontal gyrus [Brodmann area (BA) 6–1240 voxels, peak voxel at x = 0, y = −7, z = 67; Z = 5.09, pFWE = 0.01 corrected for multiple comparisons] that extended bilaterally to the supplementary motor cortex and the paracentral lobule. *Post hoc* two-group comparisons (see Table 3 for details) revealed that the GMV of this large voxel cluster encompassing these three brain regions was greater in the TTW group than in the CW group. Although not significant after correcting for multiple comparisons, the GMV of the same voxel cluster also tended to be higher in the TNTW group than in the CW group (peak voxel at x = 0, y = −12, z = 75; Z = 2.42, p = 0.002 uncorrected for multiple comparisons). Finally, CM also exhibited significantly higher GMVs in the bilateral paracentral lobule, the bilateral supplementary motor cortex and the left precentral gyrus than CW (Table 3).

**Table 3:** Significant regional grey volume differences between groups (*post hoc* two-group comparisons).

Comparison	Brain region	T^1^	P^2^	Size of cluster^3^	Coordinates (x, y, z)^4^
***Whole***-***brain exploratory analysis***					
Cisgender men > cisgender women	Paracentral lobule and SMC bilaterally and L precentral gyrus (BA 6)	5.92	0.002	1383	0 −9 67
Hormone-treated TW > cisgender women	L medial superior frontal gyrus, extending bilaterally to the SMC and paracentral lobule (BA 6)	5.29	0.023	1994	0 −8 67
***Small***-***volume correction***-***based analyses***					
Treatment-naïve TW < cisgender women	L Insula (BA 13)	4.07	0.014	828	−35 −16 13
	R Insula **(**BA 13)	4.25	0.008	437	36 −9 12
Hormone-treated TW < cisgender men	L Insula (BA 13)	4.01	0.012	67	−36 5 −14
	L Insula (BA 13)	3.79	0.031	60	−42 −7 4
Hormone-treated TW < cisgender women	L Insula (BA 13)	4.22	0.009	755	−42 −10 6
	R Insula (BA 13)	3.74	0.035	414	45 −4 3

Notes: TW = transgender women with gender dysphoria diagnosis; SMC = supplementary motor cortex; BA = Brodmann area; L = left; R = right. ^1^T value (*post hoc* tests) for the voxel of maximal statistical significance within each cluster. ^2^Statistical significance after correction for multiple comparisons; inferences were made at the level of individual voxels (family-wise error correction) and a minimum extent threshold of 30 voxels. ^3^Total number of contiguous voxels in each region above an initial cut-off of Z > 3.09. ^4^MNI coordinates of the voxel of maximal statistical significance within each cluster.

**Figure 1:**
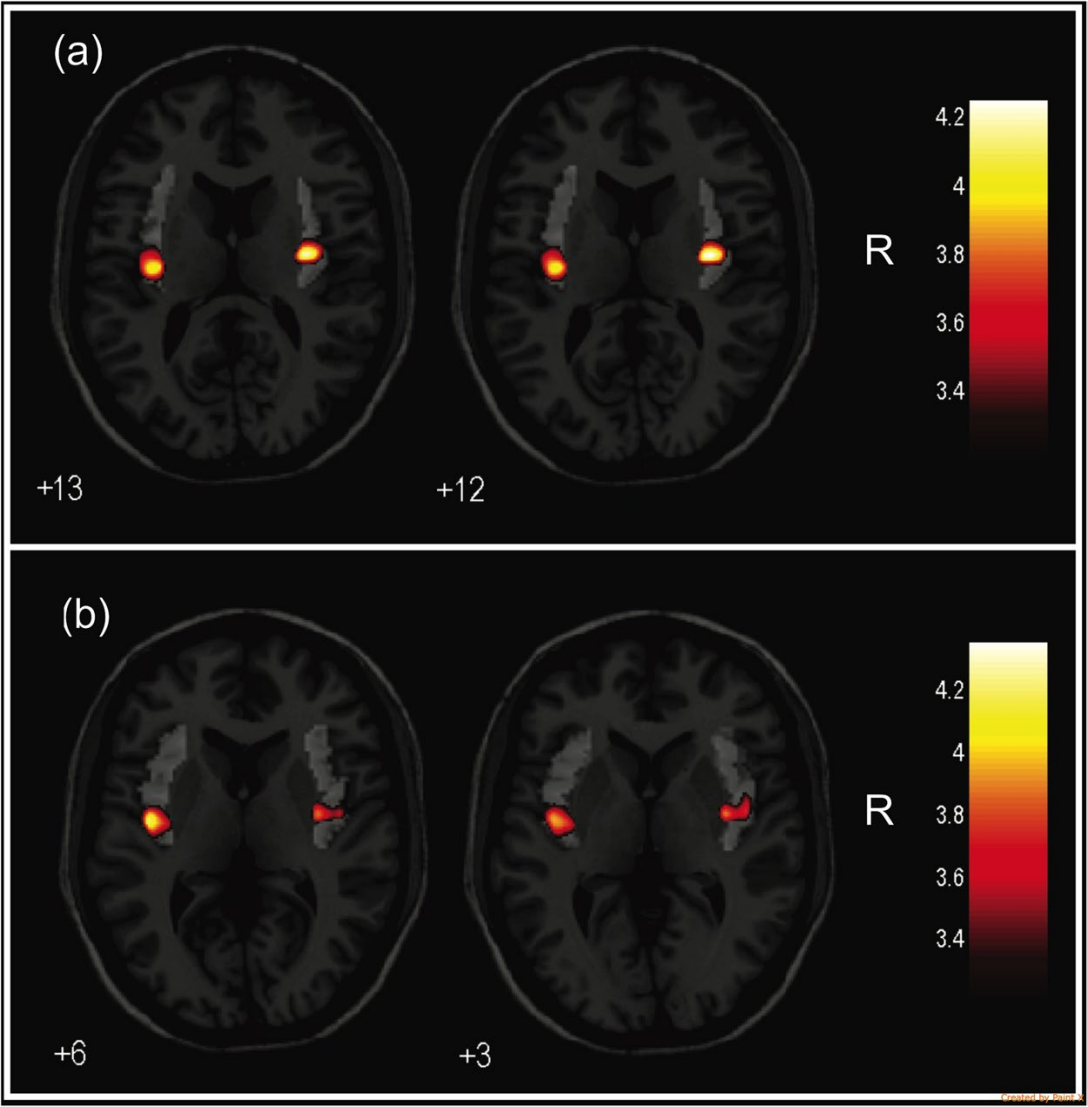
Findings showing clusters of lower grey matter volumes in the insular cortex of both hemispheres, highlighted in yellow: (**a**) in treatment-naïve TW compared with cisgender women controls and (**b**) in hormone-treated TW compared with cisgender women controls. Region-of-interest masks of the left and right insular cortex were superimposed on the images. Foci of significance are overlaid on axial brain slices spatially normalized into an approximation to the Talairach and Tournoux stereotactic atlas, and the numbers associated with each frame represent standard coordinates in the z-axis. All voxel clusters shown in the figures retained statistical significance after family-wise error correction for multiple comparisons (pFWE < 0.05, with small-volume correction over the insula) and had a minimum extent threshold of 30 voxels. Statistical details are given in Table 3. The coloured bar represents T-values. (TW = transgender women diagnosed with gender dysphoria; R = right).

We further evaluated the SPM for ANCOVA comparing regional GMVs in *a priori*-selected brain regions between groups using the SVC approach, and we observed additional significant findings in the left insula [BA 13–243 voxels, peak voxel at x = −41, y = −9, z = 6; Z = 3.78, pFWE = 0.023 corrected for multiple comparisons]. *Post hoc* two-group comparisons showed lower GMVs in the bilateral insula in both TW groups than in the CW group (Fig. 1) and a lower GMV in the left insula in the TTW group than in the CM group (Table 3). No additional GMV differences were observed in any other *a priori*-selected brain regions.

#### Regional WMV differences

No significant regional WMV differences were observed among the groups in the exploratory whole-brain analysis (pFWE < 0.05). Further evaluation of the SPM for ANCOVA comparing regional WMVs in *a priori*-selected brain regions between groups using the SVC approach revealed no statistically significant clusters (pFWE < 0.05).

## Discussion

The present VBM study investigated GMV and WMV patterns in two TW groups that were well-characterized regarding their sexual orientation and non-conformity to their sex assigned at birth from an early age. The TNTW and TTW subjects were recruited before beginning CHT and at least one year after beginning CHT, respectively, which allowed us to differentiate brain volume findings related to TW or CHT.

The lower total brain volume in CW observed here is consistent with a previously reported profile of sexual differences in the brain^10^. The finding that the three other groups presented total brain volumes larger than the CW group indicates that these brain volume differences are congruent with the sex assigned at birth in both TW groups^27^. Additionally, no differences in total brain volume were found between the TTW group and the TNTW and CM groups in the present cross-sectional study, although overall effects of CHT on reductions in total intracranial volume (TIV) and total GMV have been observed in other longitudinal MRI studies of TW undergoing CHT^30,31^.

Regarding regional GMV, we found lower GMV bilaterally in the insula in both TW groups, in contrasts to the results reported by Savic and Arver (2011), who found higher GMV in the right insula of the TW group relative to controls^20^. Likewise, Zubiarre-Elorza *et al*. (2013) verified greater cortical thickness in the right insula in TNTW than in CM controls^23^. Another MRI study conducted by Zubiarre-Elorza *et al*. (2014) reported decreased right insular volumes in TW undergoing CHT^31^. Our finding in the insula is unlikely directly due to CHT because the lower GMV of the insula was observed for both TW groups compared with the CW group. Furthermore, brain volume and cortical thickness are widely considered as complementary brain measurements^32,33^.

Manzouri *et al*.^34^ recently conducted a morphological and functional MRI study including treatment-naïve transgender men - *female sex assigned at birth* - and cisgender controls. The GMV differences found in the transgender men group followed patterns related to the sex assigned at birth. However, the authors also demonstrated weaker functional connections from the pregenual anterior cingulate to the insular cortex and to the temporo-parietal junction in the transgender men than in controls. These findings also suggest that transgender men differ from cisgender people with respect to the own-body image neural network, which may be a neurobiological substrate related to transgender men^34^. Similarly, Savic and Arver (2011) and Simon *et al*. (2013) suggested that regional GMV differences detected in transgender people emphasize the brain regions related to the body perception network^20,21^.

It is worth mentioning that an important diagnostic criterion for gender dysphoria is the distress that accompanies the incongruity between the body and gender identity, as the secondary sexual characteristics do not belong to the gender with which one identifies^35^. Decreased insular volumes have been reported in depressed subjects with melancholic symptoms^36^, and these changes in insular and prefrontal cortical volumes may be specifically associated with the manifestation of psychotic symptoms in major depressive disorder^37^. The insula is related to all subjective sensations and is the possible foundation of interoception^38^, including body awareness^39^. Furthermore, the insula connects the distinct functional systems involved in processing emotions, sensory-motor skills and cognition^40^. Two studies have evaluated functional connectivity in transgender people and found (i) a pattern of neural connectivity that inferred suffering due to incongruity between sex assigned at birth and gender identity^41^, and (ii) that the connectivity between the right insula and the somatosensory cortex was negatively related to ratings on a well-being scale in regard to gender dysphoria^42^. Another study examined brain structural connectivity and observed unique differences in regional network efficiency in the insular area in trans people^43^. These observations, together with our findings of reduced GMVs in the insula in both hemispheres in two independent TW groups, suggest that such regional GMV differences could be characteristics associated with TW. Furthermore, these alterations in the insula could be related to the neural network of body perception and reflect the distress that accompanies gender dysphoria. The hypothesis that insular volume variations in TW individuals reflect the distress of these individuals should be considered in future studies including large TW samples by evaluating significant correlations between GMV and symptom severity ratings. In addition, it is well known that the symptoms of gender dysphoria improve with CHT^35^. As a consequence of symptom relief, the TTW group could present a greater insular volume than the TNTW group. However, this possibility was not verified in the current study, which suggests that GMV differences in the insular cortex in both TW groups may be related to a more general “transgender trait”.

One important difference between our VBM study and the study performed by Savic and Arver (2011) is that the latter investigated non-androphilic TW (sexually attracted to females, both males and females, or neither gender)^20^ according to the Blanchard classification, which is based on the sex assigned at birth of TW^44^, while our study investigated androphilic TW (sexually attracted to males). Therefore, one might speculate that the differences observed in the insular cortex could be linked to the sexual orientation of TW. It should be noted, however, that sexual orientation and gender identity are distinct phenomena. For this reason, overlapping VBM findings associated with these two conditions are highly speculative. Another VBM study assessing hetero- and homosexual CM did not identify any regional GMV differences between groups. Homosexual CW presented lower GMV in the perirhinal cortex than heterosexual CW^45^. Considering these speculative findings, further MRI studies comparing androphilic TW with non-androphilic TW are required to reveal further information on this topic.

We also detected lower regional GMV in a large portion of the posterior-superior frontal cortex in the CW controls than in both TW groups and the CM controls. Because our statistical approach was primarily designed to identify regional GMV variations that could be related to gender dysphoria, direct *post hoc* two-group comparisons between the cisgender controls in our study should not be considered as comprehensive indicators of a predominant profile between the two control groups regarding regional brain volume differences. In addition, the lower GMV detected in the posterior-superior frontal cortex in the CW group is consistent with previously described regional patterns of sexual differences of the human brain^10,46^. Significantly higher GMV in the posterior-superior frontal cortex was also detected only in the TTW and CM groups relative to the CW group. Conversely, the difference in regional GMV in this frontal region in the TNTW group relative to the CW group was not significant after correcting for multiple comparisons. These results of the present study may indicate that this brain region in TNTW tends to present female characteristics, although this conclusion should be interpreted with caution.

Based on the abovementioned interpretation, the finding of higher GMV in the posterior-superior frontal cortex in the TTW group might seem contradictory, given the overall feminizing effects of CHT. Nevertheless, previous studies have shown increased GMV in several cortical regions in postmenopausal women under oestrogen replacement therapy^47^, including higher GM density in paracentral and precentral areas^48^ and in the superior frontal gyrus^46^. Such findings are believed to reflect the influence of hormonal treatment on brain neuroplasticity.

Overall, our results suggest that the impact of CHT on brain volume may vary across regional and global levels, as we found no difference in total brain volume between the two TW groups, despite the fact that the TTW had undergone prolonged use of sex steroids. A similar study conducted by Hoekzema *et al*. (2014) revealed that the neuroanatomical characteristics of treatment-naïve transgender adolescents and transgender adolescents undergoing CHT corresponded to those of individuals of the same sex assigned at birth.

Such results are in contrast to the findings of previous studies of TW using repeated MRI measurements before and during CHT. Hulshoff Pol *et al*. (2006) reported reduced global brain volumes in TW after four months of CHT towards the volumes expected for CW^30^. Similar to our TW sample, Zubiaurre-Elorza *et al*. (2014) investigated TW after at least six months of hormonal treatment and reported reduced cortical thicknesses and volumes of subcortical structures in the right hemisphere (specifically in the pallidum and the thalamus) and an enlarged ventricular system^31^. A recent study showed increased ventricle and reduced right hippocampal volumes in TW after four months of CHT^49^. These differences between our results and those of previous investigations may be because the present study employed a cross-sectional design, while the previous studies were longitudinal investigations of the same individuals and, thus, may have been more sensitive to detecting CHT-related global volume changes over time in TW.

Our findings, as well as the results of all previous brain imaging studies that have evaluated the influence of CHT, suggest that the effects of CHT on brain volume may differ at global and regional levels and may vary across brain structures. Moreover, the different results obtained for the two TW groups in our study suggest that the association of brain volumes with gender dysphoria and CHT may be complex, and furthermore, the direction of associations of brain volume with these two dimensions may vary.

Conflicting VBM-based findings among MRI studies of TW regarding subcortical GM structures have been reported to date. In the first VBM study to evaluate TW, Luders *et al*. (2009) identified a greater GMV in the right putamen^19^. Through SVC-based analyses, Savic and Arver (2011) also reported that TW subjects exhibit a greater GMV in the right putamen than control subjects; conversely, ROI-based measurements indicated reduced volumes of the putamen in TW relative to controls^20^. In the present study, the SVC-based analysis did not reveal significant group differences in voxels located specifically in the basal ganglia (although relatively larger clusters of lower GMV in the insula extending towards the right putamen were observed in both TW groups, as shown in Fig. 1). Finally, the paucity of between-group differences in the WM analyses in our study suggests that brain volume differences related to the effects of both TW and CHT predominate in the GM compartment of the brain.

Some methodological limitations of our study must be considered. First, because this study employed a cross-sectional design, it was not possible to determine which changes were pre-existing and which resulted from CHT. Second, our sample size can be considered small (n = 80) and may not confer confidence in the results obtained, although the sample size was similar to those in previous MRI studies in this field^20,23,43^. Third, variability existed in the duration of CHT among individuals in the TTW group. Finally, the stage of the menstrual cycle of the CW was not considered at the time of MRI scanning, which may be a relevant factor considering that brain structures are influenced by hormonal physiological variations^50^.

In conclusion, significant regional brain volume differences in this VBM study were detected in TW compared with cisgender controls. In TTW, the observed brain volume changes suggested a possible influence of CHT on brain neuroplasticity^46,47^. Finally, we present a novel finding of GMV alterations in the insula in the two independent TW groups, which may be a characteristic of TW. These alterations in the insula could be related to the neural network of body perception and may reflect the distress that accompanies gender dysphoria.

## Methods

### Sample

Inclusion criteria for all TW were as follows: (a) absence of changes (except for changes resulting from the use of CHT by TTW) in secondary male sex characteristics according to the Marshall and Tanner criteria^51^, as evaluated by a physical examination performed by endocrinologists at HC-FMUSP; (b) a history of non-conformity to birth sex since the early stages of child development; (c) compatibility with gender dysphoria diagnostic criteria as per the DSM-5 and based on semi-structured interviews conducted by three mental health professionals (one psychiatrist and two psychologists); and (d) exclusive sexual attraction to males according to the Kinsey scale of sexual orientation^52^, as ascertained by actively asking TW about their sexual attraction (i.e., reports on desire, thoughts, fantasies and/or sexual activity with men, including romantic ideas). Specific inclusion criteria for the TNTW group were as follows: the absence of any hormonal treatment history and the expressed desire to be treated with CHT. The procedures performed in TNTW included evaluations of the plasma levels of oestradiol, follicle-stimulating hormone, luteinizing hormone, testosterone and free testosterone on the day of MRI. For TTW, the specific inclusion criterion was currently receiving CHT for at least one year, as recommended by the Endocrinology Service at HC-FMUSP. This treatment regimen consisted of the administration of one or two 0.625-mg conjugated oestrogen tablets and one 50-mg cyproterone acetate pill per day^53^. Blood hormone levels of the TTW group were evaluated every semester. Hormone dosages of all TW were calculated in the Hormones Laboratory and Molecular Genetics LIM-42 at HC-FMUSP via immunofluorometric assays using AutoDELFIA kits (PerkinElmer Inc., Waltham, MA, USA). For the cisgender controls, the inclusion criterion was heterosexual orientation, as determined by the Kinsey scale of sexual orientation^52^.

Exclusion criteria based on the Structured Clinical Interview for DSM-IV Axis I Disorders (SCID)^54^ for all groups included a history of psychiatric disorders and prior use of psychopharmacological agents. Additional exclusion criteria included the presence of medical conditions or neurological disorders that could affect the central nervous system, contraindications to MRI, major medical illnesses, and substance abuse.

The study sample included 80 individuals between 18 and 49 years of age divided into the following four groups (n = 20 each): TNTW, TTW, CW and CM (cisgender individuals as healthy controls). The selection of TNTW was based on a waiting list of those individuals who identified themselves as transgender women and who were awaiting treatment at HC-FMUSP, contacts with non-governmental organizations that provide assistance to transgender people and contacts with mental health expert clinicians who practice in São Paulo and who were able to refer patients for the study. The TTW group was selected from among those patients who were already receiving treatment at HC-FMUSP. Healthy voluntary controls were verbally invited among community and staff members at HC-FMUSP.

All participants were fully informed about the study and signed a consent form. This study followed the principles of the Declaration of Helsinki and was approved by the Ethics Committee at the Medical School Hospital, University of São Paulo (HC-FMUSP), Brazil.

### Image acquisition and processing

All MRI scans were acquired using a 1.5 T Siemens Espree system (Siemens, Erlangen, Germany) at the Institute of Psychiatry, HC-FMUSP. Morphological brain data were acquired using a T1-weighted magnetization-prepared rapid gradient echo sequence (MPRAGE) with the following parameters: TR = 2,400 ms; TE = 3.65 ms; NEX = 1; field of view (FOV) = 240 mm; flip angle = 8o; matrix = 192 × 192 pixels; slice thickness = 1.2 mm (no gap between slices); and voxel size = 1.25 × 1.25 × 1.2 mm, resulting in 160 slices covering the whole brain.

All images were reviewed by a neuroradiologist with the purpose of identifying artefacts during image acquisition and the presence of silent gross brain alterations. VBM processing was performed using the Statistical Parametric Mapping (SPM) program, version 8 (Wellcome Trust Centre of Neuroimaging, London, United Kingdom), implemented in MATLAB R2012a (MathWorks, Sherborn, Massachusetts). First, all anatomical images were reoriented; the mm coordinate of the anterior commissure matched the x y z origin (0, 0, 0), and the orientation approximated Montreal Neurological Institute (MNI) space. The images were then segmented into GM, WM and cerebrospinal fluid (CSF) partitions using the unified segmentation procedure described by Ashburner and Friston^55^. The Diffeomorphic Anatomical Registration Through Exponentiated Lie Algebra (DARTEL) algorithm was then used to spatially normalize the segmented images. This procedure maximizes sensitivity and the accuracy of localization by registering individual structural images to an asymmetric T1-weighted template derived from participants’ structural images rather than a standard T1-weighted template based on a different sample^56^.

These fully normalized images were resliced through trilinear interpolation to a final voxel size of 1.5 × 1.5 × 1.5 mm³. An additional “modulation” step consisted of multiplying each spatially normalized GM and WM image by its relative volume before and after normalization. This step ensured that the total amount of GM and WM in each voxel was preserved. Finally, the resulting GM and WM images were smoothed using an 8-mm isotropic kernel at full width at half maximum (FWHM) to ensure normal distribution of the data as required by subsequent statistical parametric tests.

### Statistical analysis

#### Demographical and clinical data

The Statistical Package for the Social Sciences (SPSS) for Windows, version 18.0 (SPSS, Chicago, IL, USA) was used. All statistical tests were conducted with a 5% significance level. Age and education in years were compared between groups using the Kruskal-Wallis test. Differences in hormonal levels between the TW groups were verified by the Mann-Whitney *U*-test. Fisher’s exact test was used to evaluate handedness.

#### Neuroimaging data

Total GM, WM and CSF volumes were calculated by the MATLAB get_totals script (http://www.cs.ucl.ac.uk/staff/g.ridgway/vbm/get_totals.m) implemented for SPM8 using the segmented images on the native space for each subject. Between-group differences in total GM, CSF and WM volumes, total tissue volume (TV, calculated as GM + WM), and TIV (calculated as GM + WM + CSF) were tested with analysis of variance (ANOVA) using SPSS. Age and years of education were included as covariates in such comparisons.

Regarding the VBM analyses, we initially performed exploratory voxel-wise comparisons of regional GMVs and WMVs among the 4 groups across the whole brain using an analysis of covariance (ANCOVA) model in SPM (*F* test). Given the between-group differences in years of education, this variable was included as a covariate in such comparisons. Although the mean age was not significantly different in between groups, age was also entered as a further confounding covariate. The age range of the overall sample was relatively wide (from 18 to 49 years), and in such situations, previous VBM studies have shown that it is appropriate to include age as a confounding covariate in between-group comparisons^57^. The measures of total GM or WM in the brain were also entered as confounders in the respective ANCOVA. Only voxels with values above an absolute threshold of p = 0.05 for differentiating GM and WM from other tissues were included in the analyses. Significant ANCOVA findings were followed up with *post hoc* tests for two-group comparisons (*t* tests). The resulting statistics were thresholded at a p < 0.001 (uncorrected for multiple comparisons) level of significance (Z = 3.09) and displayed as SPMs in standard anatomical space. Only clusters with a minimum of 30 voxels were reported (30 voxels correspond to approximately 100 mm^3^; this threshold has been used in previous studies^21,58^ and offers an easy-to-understand size scale for the reader). Findings in these exploratory analyses were considered significant if they survived family-wise error (FWE) correction for multiple comparisons over the whole brain (p_FWE_ ≤ 0.05).

Subsequently, each statistical map was inspected for the presence of clusters of significant differences in brain regions where volumetric differences had been predicted *a priori* through the SVC method, with the purpose of constraining the total number of voxels included in the analysis. Each GM region was spatially delimited by applying masks onto the SPMs, based on the anatomical volumes of interest that are available within the automatic anatomical labelling SPM toolbox (http://www.gin.cnrs.fr/AAL). All the anatomical masks were used separately in each hemisphere. SVC was applied over the following brain regions: the angular gyrus, the insula and the putamen. These regions were predicted *a priori* to show differences in VBM studies of TNTW^19–21^. For SVC-based analyses of WM regions^18^, ROIs over the corpus callosum (body, genu, and splenium) were defined based on the JHU DTI-based WM atlases^59–61^. Findings for these hypothesis-driven, SVC-based analyses were reported as significant if they survived FWE correction for multiple comparisons (p_FWE_ ≤ 0.05) over the search volume of each ROI, with a cluster size threshold of 30 contiguous voxels.
